# Effects and mechanism of puerarin on lactation of postpartum hypogalactia mice

**DOI:** 10.22038/ijbms.2025.81350.17611

**Published:** 2025

**Authors:** Ya-jie Yang, Bing-qing Liu, Yang Jiang, Man-yu Zhou, Re-yi-la Tuerxun, Hong-shuang Liu, Yan Liao

**Affiliations:** 1 School of Chinese Medical, Beijing University of Chinese Medicine, Beijing, 102446, China; 2 Traditional Chinese Medicine Hospital of Xinjiang Uyghur Autonomous Region, Xinjiang, 830092, China

**Keywords:** Breast feeding, Domperidone, Galactogogues, Phytoestrogens, Prolactin

## Abstract

**Objective(s):**

Insufficient breast milk supply is a common reason cited for discontinuing breastfeeding prematurely. Natural galactagogues offer promise as a solution for mothers with low milk production. This study aimed to explore puerarin’s potential effects and underlying mechanism on lactation of postpartum hypogalactia mice.

**Materials and Methods:**

Postpartum mice were randomly assigned to five groups: control group, agalactosis model group, domperidone group (3.5 mg/kg), low dose puerarin group (18 mg/kg), and high dose puerarin group (72 mg/kg). The effects of puerarin on postpartum hypogalactia mice were evaluated by lactation indicators and pathological morphology. Related hormones and prolactin receptor (PRLR)/Janus kinase 2 (JAK2)/signal transduction and activator (STAT) 5 signaling pathway were also measured.

**Results:**

Puerarin significantly improved lactation yield and stimulated mammary gland development in postpartum hypogalactia mice. Additionally, puerarin increased the expression levels of β-casein, fatty acid synthase (FAS), and glucose transporter 1 (GLUT1). Mechanically, puerarin stimulated secretion of prolactin (PRL), estradiol (E2), and progesterone (P4) in agalactosis mice. Puerarin also substantially increased PRLR, JAK2, and STAT5a expression levels in postpartum hypogalactia mice.

**Conclusion:**

This study suggested that puerarin may promote lactation by stimulating PRL secretion and activating the PRLR/JAK2/STAT5 signaling pathway.

## Introduction

Human milk is widely recognized as the most natural and dependable source of nutrition for newborns (1). Within the first six months, human milk provides infants with the nutrients and bioactive components to promote healthy gastrointestinal development, neural growth, and immune protection (2-4). Meanwhile, breastfeeding offers advantages to mothers, such as faster uterine involution, weight loss, and reduced risks of cardiovascular disease, breast cancer, ovarian cancer, and postpartum depression (5). Consequently, the World Health Organization recommends exclusive breastfeeding for at least six months after childbirth (1). Unfortunately, in China, the exclusive breastfeeding rate within the first six months is a mere 29.2% (6). As stated by the American Academy of Pediatrics, breastfeeding must be treated as a public health concern that requires urgent attention (7). 

Insufficient milk supply is commonly cited as the reason for ceasing breastfeeding prematurely or low breastfeeding rate (8). Generally, methods for increasing human milk production could be divided into non-pharmacologic and galactagogues (9). Galactagogues are substances believed to assist with milk initiation, maintenance, or augmentation (10). They can be pharmacological (e.g., domperidone) or natural (e.g., foods or plants) (11). Nevertheless, pharmacological galactagogues such as domperidone may not be suitable for all women, particularly those with a history of cardiac diseases (12). In recent years, natural galactagogues have gained more attention as a possible solution to insufficient milk supply. 

**Figure 1:**
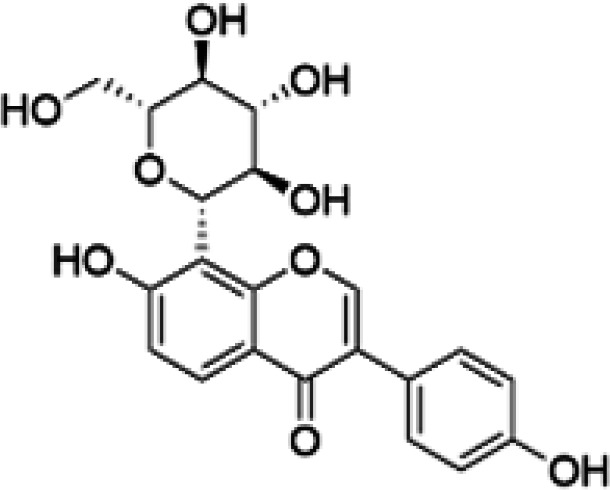
Chemical structure of puerarin

Pueraria lobata, an edible and medicinal homologous plant used in China for thousands of years (13), is employed to treat various ailments such as fever, headache, backache, diarrhea, dysentery, and menopausal symptoms (14, 15). In Thailand, residents commonly consume P. lobata to enlarge the breasts and improve skin condition (16). Puerarin (7,4’-dihydroxy-8-C-glucosylisoflavone) (Figure 1), a phytoestrogen, is the primary bioactive ingredient in P. lobata (14). A relevant study showed that puerarin may enhance mammalian mammary development and elevate prolactin (PRL) levels by acting on the pituitary (17). Our preliminary research revealed that a low dose of puerarin has a beneficial effect on promoting lactation in postpartum normal mice (18). However, it remains unclear about the effects of puerarin in animal models of postpartum hypogalactia. Therefore, this study aimed to elucidate the potential effects of puerarin on milk yield and quality in postpartum hypogalactia mice, as well as the underlying mechanism.

## Materials and Methods

### Chemicals and reagents

Puerarin (purity ≥ 98%), domperidone (purity ≥ 98%), and tissue fix solution (4% formaldehyde solution, neutral buffered) were purchased from Yuanye Bio-Technology Co., Ltd. (Shanghai, China). Pentobarbital sodium was purchased from Honghu Lianhe Huagong Chanpin Co., Ltd. (Beijing, China). Methyl alcohol was purchased from the Liuyi chemical plant (Beijing, China). Bromocriptine mesylate tablet was purchased from Gedeon Richter Plc. (Shanghai, China). Haematoxylin and Eosin (H&E) staining kit and tween-20 were purchased from Solarbio Science & Technology Co., Ltd. (Beijing, China). PRL ELISA kit was purchased from Cloud-Clone Corp. (Wuhan, China). Estradiol (E2) and progesterone (P4) ELISA kits were purchased from Meilian Biotechnology Co., Ltd. (Shanghai, China). Maker was purchased from Proteintech-CN Group, Inc. (Wuhan, China). Signal transduction and activator (STAT) 5a antibody and primary antibodies of prolactin receptor (PRLR) and β-casein were purchased from BIOSS Biological Technology Co., Ltd. (Beijing, China). Electrochemiluminescence (ECL) reagent and phosphate buffer saline dry powder were purchased from Absin Bio-Technology Co., Ltd. (Shanghai, China). 5×Loading buffer was purchased from Biorigin Inc. (Beijing, China). Poly (vinylidene fluoride) (PVDF) membrane (0.45 μm) was purchased from Merck Millipore Co., Ltd. (Beijing, China). All other chemicals and reagents were obtained from Servicebio Technology Co., Ltd. (Wuhan, China).

### Animals

Forty-five female and fifteen male specific pathogen-free Kunming mice, aged 7–8 weeks and weighing 34 ± 2.26 g, were supplied by the SPF (Beijing) Biotechnology Co., Ltd. (license No. SCXK (Jing) 2016-0002). The mice were kept under specific pathogen-free conditions with controlled temperature (25 ℃) and humidity (40–70%) at the Beijing Academy of Chinese Medicine. They were allowed to access standard mouse feed and water ad libitum. The animal experiments were approved by the Experimental Animal Management Committee of Beijing Academy of Chinese Medicine (Ethics approval number: BUCM-4-2019103101-4031) and conducted following relevant guidelines and regulations.

### Assessing estrous cycle and mating

The estrous cycle of the mice was monitored by using vaginal smears. Each day at 9:00 AM, a cotton swab moistened with normal saline was inserted into the vagina of the restrained mice. The swab was rotated to collect vaginal secretions and then removed. The collected vaginal secretions were smeared on a glass slide. The slides were left to air-dried and then fixed with a 4% formaldehyde solution for three minutes. Following fixation, the slides were stained by H&E kits and sealed with neutral balsam. Finally, the slides were examined under a microscope to determine the estrous stage of the mice. Estrus was characterized by numerous cornified epithelial cells and few nucleated epithelial cells.

Female mice in estrus were paired with males in a 3:1 ratio (female: male) at 3:00 PM. On the second morning at 9:00, the females were checked for the presence of ivory or light yellow vaginal plugs, indicating successful mating. The mated females were then housed individually until delivery. 

### Experimental design and treatments

**Figure 2:**
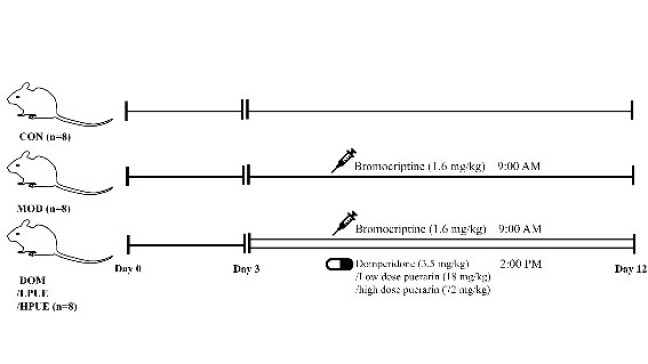
Animal Experimental Protocol

Forty dams delivering within 24 hr were selected for the experiments. Each litter was adjusted to 8 pups. Post-delivery, the mice were randomly divided into five groups: control group (CON, n=8), agalactosis model group (MOD, n=8), domperidone group (DOM, n=8), low-dose puerarin group (LPUE, n=8), and high-dose puerarin group (HPUE, n=8) (Figure 2). 

The delivery day was recorded as day zero. From day 3 post-delivery, all groups except the control group received bromocriptine (1.6 mg/kg) intragastrically at 9:00 AM for ten days to establish the agalactosis model. The dosage was based on a previous study (19).

While establishing the model, the treatment groups received intragastric administration of domperidone (3.5 mg/kg), low-dose puerarin (18 mg/kg), or high-dose puerarin (72 mg/kg) at 2:00 PM for 10 days. The dosage was determined based on a previous study (20) and a guideline (21). The control and agalactosis model groups received an equal volume of normal saline. 

### Data collection on lactation indicators

*Weight change of pups and postpartum mice*

The weight measurements were taken on the 3^rd ^and 12^th ^day to determine the initial and final weights, respectively. The weight changes of pups and postpartum mice were calculated as the final weight minus the initial weight.

*Lactation yield measurement*

From day 3, the total litter weight (W1) was measured at 9:00 AM daily. Pups and mothers were separated for five hours, and the litter weight was measured again (W2). Pups were returned to their mothers to suckle for one hour, and the litter weight was re-recorded (W3). The hourly milk yield was calculated as the weight difference during suckling (W3 – W2) plus the average basal metabolic rate for one hour ((W1 – W2) / 5):

The average milk yield (g/h) = (W3 – W2) + (W1 – W2) / 5 (19)

This measurement continued for 10 days.

### Sample collection and measurement

On day 12, the parental mice were fasted for 12 hr starting at 7:00 PM. On day 13, mice were anesthetized with 1% pentobarbital sodium (10 ml/kg, intraperitoneally), and the blood samples were collected by puncturing the inner canthus of mice with a hard capillary glass tube. The blood sample, approximately 300 μl, was set for one hour at room temperature and then centrifuged at 4000 rpm/min for 15 min. Serum was collected and stored at -80 °C. The third pair of gland tissue in the hypogastrium of the female mice were separated and weighed. A portion of the gland tissue was placed in a 4% formaldehyde solution, while the other part was stored at -80 °C. The mammary gland index was calculated as the ratio of mammary gland weight to body weight on the last day of the experiment.

### Histopathological analysis

The breast tissues were removed from the 4% formaldehyde solution and promptly shaped. Subsequently, the tissues underwent dehydration using a series of ethanol, clarification in xylene, embedding in paraffin, slicing, and finally staining with H&E. The pathological morphology of the breast tissue was observed and photographed under an optical microscope (Nikon DS-U3, NIKON, Japan).

### Biochemical analyses

Mammary gland tissues were removed from the refrigerator, fragmented, ground, and centrifuged at 12,000 rpm/min for 15 min to obtain the supernatants. Concentrations of PRL, E2, and P4 in serum and PRL in mammary tissue were measured using ELISA kits according to the provided instructions. The optical density at 450 nm was measured using a microplate reader (iMark20675, Bio-Rad, USA).

### Western blotting analysis

Expression of related proteins was determined using Western blotting. Mammary tissue protein samples were retrieved from the refrigerator. The protein concentrations were quantified using bicinchoninic acid protein assay kits. Subsequently, the proteins were separated using sodium dodecyl sulfate-polyacrylamide gel electrophoresis (SDS/PAGE) and transferred onto PVDF membranes. The PVDF membranes were blocked with 5% non-fat dried milk in tris-buffered saline containing Tween-20 (TBST) for one hour, followed by overnight incubation with the primary antibody at 4 °C. Next, the membranes were incubated with a secondary antibody for one hour at room temperature. Finally, the membranes were visualized using an ECL reagent and exposed to a gel imaging system (Che miDoc Touch, Bio-Rad, USA). 

### Statistical analysis

Statistical analysis was performed using SPSS 23.0 (SPSS Inc., Chicago, IL, USA). Group differences were analyzed by one-way analysis of variance (ANOVA), followed by the least significant difference (LSD) test. The results were expressed as mean ± standard deviation (SD). When heterogeneity of variance was observed, group differences were analyzed using the Kruskal-Wallis test, followed by Bonferroni correction, and the results were expressed as the median and interquartile range (IQR). Statistical significance was considered at P<0.05. Bar charts were created using GraphPad Prism9 (GraphPad, Inc., San Diego, CA, USA).

## Results

### Puerarin improves the lactation function of postpartum hypogalactia mice

**Figure 3:**
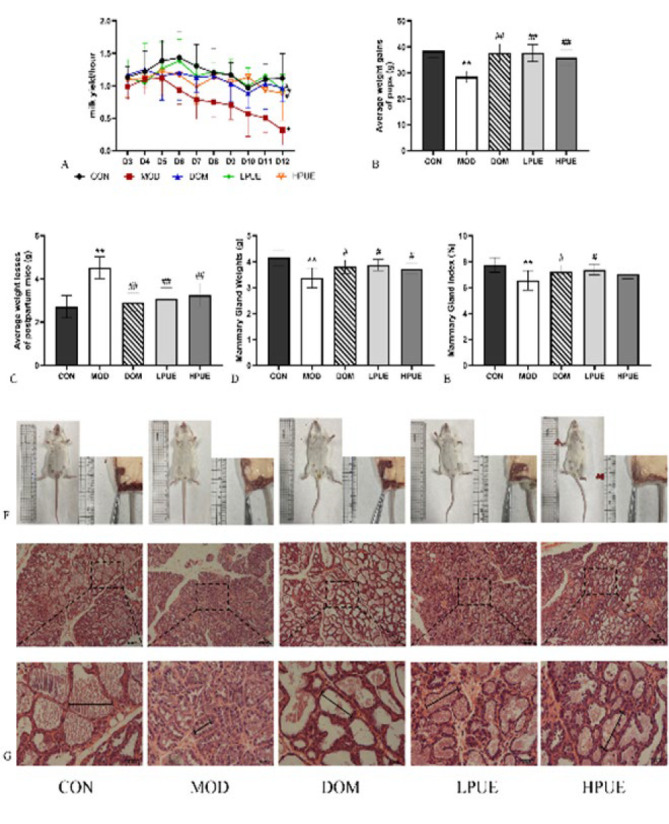
Puerarin effects on lactation of postpartum hypogalactia mice

The lactation function of mice can be assessed by measuring the lactation yield. As shown in Figure 3A, there was no significant difference in the average milk yield between each group during the first four days of the experiment (*P*>0.05). However, from day 5, the average milk yield of the agalactosis model group was statistically lower than that of the control group (*P*<0.05). Meanwhile, the milk yields of the low-dose puerarin and domperidone groups were significantly higher than the agalactosis model group from day 5 (*P*<0.05). Additionally, from day 6, the average milk yield of the high-dose puerarin group was statistically higher than the agalactosis model group (*P*<0.05). Throughout the lactation, the milk yield of the control group was roughly equivalent to that of the treatment groups (*P*>0.05).

In addition to the lactation yield, the average weight gains of pups can serve as an indirect measure of the lactation function of dams. Figure 3B illustrated that the average weight gains of pups in the model group were statistically lower than that in the control group after a 10-day period (*P*<0.01). Conversely, the average weight gains of pups in treatment groups showed a substantial increase compared to the model group (*P*<0.01).

Furthermore, Figure 3C demonstrated that the average weight loss of postpartum mice in the agalactosis model group was statistically higher than in the control group (*P*<0.01). However, the average weight losses of postpartum mice in treatment groups were significantly lower than that in the model group, indicating a substantial recovery in the weight of postpartum mice (*P*<0.01).

To investigate the effects of puerarin on the morphology of the mammary gland in postpartum hypogalactia mice, we examined the mammary glands of dams (Figure 3F). Compared to the control group, the mice in the agalactosis model group were visibly thinner and exhibited a noticeable reduction in the subcutaneous distribution area of the mammary gland. Conversely, the mice in the treatment groups appeared plumper and had a larger mammary gland subcutaneous distribution area. Furthermore, we calculated the mammary gland weights and index (Figure 3D, 3E). The mammary gland weights and index of agalactosis model mice were significantly decreased (*P*<0.01), which were substantially recovered by either supplement of puerarin or domperidone (*P*<0.05).

Histological slices were conducted to observe the pathological changes in the mammary glands. In the mammary tissue of the control group (Figure 3G), the glandular cavity was large and regular, filled with secretions, and contained numerous circular vacuoles consisting of lipid droplets. In the model agalactosis group, the mammary gland showed almost complete atrophy, characterized by small and irregular glandular cavities, thickened interstitium in the lobules, and significantly reduced secretions within the glandular cavity. After supplementation with domperidone or puerarin, the morphology of the mammary gland in treatment groups improved substantially. Compared to the model group, the low or high-dose puerarin group showed a notably larger area of breast lobules. The adipocytes and connective tissue between the breast lobules were significantly reduced, the size of glandular cavities returned to normal levels, and a great deal of secretions, along with a few lipid droplets, were observed in the glandular lumen.

### Puerarin induces the expressions of β-casein, fatty acid synthase (FAS), and glucose transporter 1 (GLUT1) in the mammary gland of postpartum mice

**Figure 4:**
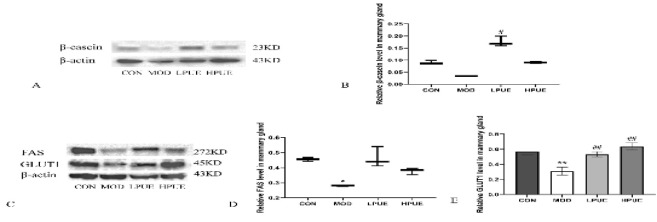
Puerarin induces the expressions of β-casein, FAS, and GLUT1 in the mammary gland of postpartum mice

To investigate puerarin’s potential in improving breast milk quality, we conducted Western blotting to determine the expressions of lactoprotein, butterfat, and lactose. Figure 4A showed that in the agalactosis model group, the expression of β-casein was reduced compared to the control group. Treatment with puerarin significantly enhanced the expression level of β-casein in milk-deficient mice (*P*<0.05) (Figure 4B). Figure 4C-4E illustrated the effects of puerarin on the expressions of FAS and GLUT1. Compared to the control group, the expression levels of FAS and GLUT1 in the agalactosis model group were significantly reduced (*P*<0.05). Puerarin treatment effectively restored the expressions of GLUT1 (*P*<0.01). 

### Puerarin stimulates PRL, E2, and P4 secretion

**Figure 5:**
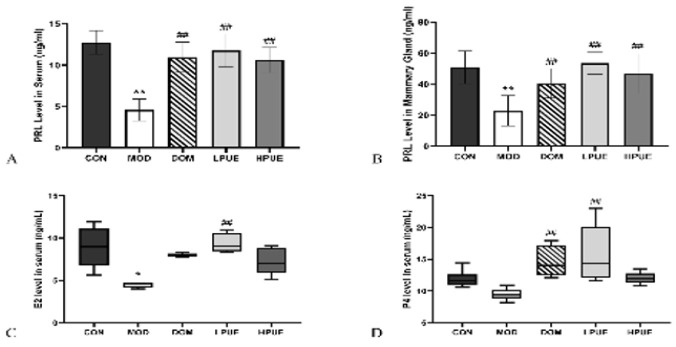
Puerarin stimulates PRL, P4, and E2 secretion

To explore the effects of puerarin on hormone levels during lactation, the concentration levels of PRL, E2, and P4 were measured using ELISA kits. Figures 5A and 5B demonstrated that the levels of PRL were statistically decreased in both serum and mammary gland circulation in the agalactosis model group compared to the control group (*P*<0.01). Interestingly, the levels of PRL were increased in serum and mammary gland circulation of treatment groups by either supplementation with puerarin or domperidone and showed a remarkable difference with the agalactosis model group (*P*<0.01). As shown in Figure 5C, the serum E2 level of maternal mice in the agalactosis model group was statistically lower than that of the control group (*P*<0.05). However, the serum E2 levels of treatment groups were recovered, especially the E2 level in the low-dose puerarin group was remarkably increased compared to that of the model group (*P*<0.01). Figure 5D reveals that the serum P4 level in the agalactosis model group was lower than in other groups, with a statistical difference observed compared to the low-dose puerarin group and domperidone treatment group (*P*<0.01).

### Puerarin activates the PRLR/ Janus kinase 2 (JAK2)/STAT5 signaling pathway

**Figure 6:**
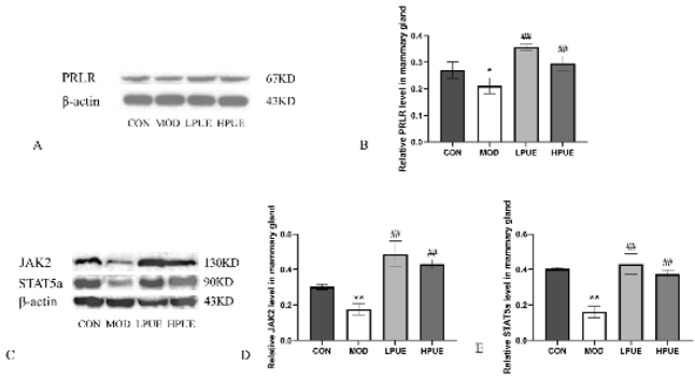
Puerarin activates the PRLR/JAK2/STAT5 signaling pathway

**Figure 7:**
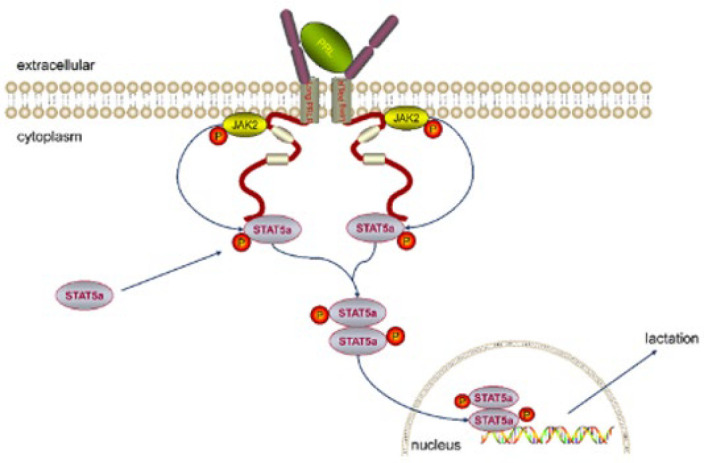
Puerarin promotes lactation by activating the PRLR/JAK2/STAT5 signaling pathway

To explore the mechanism underlying the promotion of lactation by puerarin, the expressions of PRLR, JAK2, and STAT5a were measured via Western blotting. As Figure 6A shows, in contrast with the control group, the expression of PRLR in the agalactosis model group was significantly reduced (*P*<0.05) (Figure 6B). Conversely, the expression level of PRLR in treatment groups, especially in the high dose puerarin group, was significantly higher than that in the agalactosis model group (*P*<0.01). As shown in Figure 6C-6E, the protein expression levels of JAK2 and STAT5a in the agalactosis model group were substantially decreased compared to the control group (*P*<0.01). However, supplementation with either a low or high dose of puerarin resulted in a statistically significant increase in the levels of JAK2 and STAT5a (*P*<0.01). Collectively, these data demonstrated that puerarin could promote lactation via the PRLR/JAK2/STAT5 signaling pathway (Figure 7).

## Discussion

The benefits of breastfeeding are acknowledged both for mothers and infants in the short and long term. However, many mothers encounter challenges with insufficient milk supply when they desire to breastfeed their children. The natural galactagogue as a feasible method attracts increasing attention (11). P. lobata is an edible and medicinal homologous plant with the major bioactive ingredient puerarin. Relevant research highlighted the potential of puerarin in managing lactation insufficiency (17). In light of this, we conducted this study to explore whether puerarin could serve as a new natural galactagogue for promoting lactation and, if so, to elucidate its underlying mechanism of action. 

The average milk yield is objective evidence for measuring lactation function. Like the effects of domperidone, the average milk yield of postpartum mice in the low or high-dose puerarin group was significantly higher than that of the agalactosis model group. Additionally, histological examinations of the mammary gland revealed that puerarin limited the atrophy and promoted the hyperplasia of the alveolar. Notably, low-dose puerarin showed better effects than high-dose puerarin, although the difference between the two doses was insignificant. These results indicate that puerarin may have a bidirectional regulatory effect as a phytoestrogen. Similarly, relevant research suggested that genistein regulates mammary epithelial morphogenesis as a stimulator and inhibitor, depending on its concentration (22). Our previous study also demonstrated that the low dose of puerarin can promote lactation in normal postpartum mice, while a high dose does not significantly promote lactation (18, 23).

In addition to promoting lactation yield, puerarin may enhance milk quality. Casein is the major component of human milk protein, providing crucial amino acids, trace elements (calcium and phosphorus), and bioactive proteins for infant development (24, 25). Fats are the important energy sources in mature milk that meet about 50% of the energy requirements for infants (26). The FAS plays an indispensable role during the synthesis of fats (27). Lactose is the main carbohydrate in human breast milk (28). It is responsible for providing energy, maintaining osmotic pressure, and promoting the absorption of bioactive components (25, 26). GLUT1, one of the principal glucose transporters in mammary epithelial cells, is hypothesized to be a rate-limiting factor for lactose synthesis (29). In this study, the expression levels of β-casein, FAS, and GLUT1 in the agalactosis model group were remarkably decreased. However, puerarin treatment resulted in varying degrees of recovery in the expressions of β-casein, FAS, and GLUT1. 

Lactation is a complex physiological process that relies on adequate mammary gland development, normal hormone levels, and regular and effective milk removal to ensure successful breastfeeding (12). Reproductive and metabolic hormones, including PRL, E2, and P4, are essential for mammary gland development and lactation (12, 30). Among these hormones, PRL, a neuroendocrine hormone primarily synthesized by the anterior pituitary lactotrophs, is particularly crucial (30). Chen *et al*.’s study has confirmed that UEC4-1, a peptide from Oyster Hydrolysates, can up-regulate PRL synthesis and promote lactation (31). Another research suggests that Hemerocallis citrina Baroni promotes lactation through the PRL signaling pathway in the mammary gland (32). Therefore, this study examined the levels of PRL in both serum and mammary gland circulation in an agalactosis model group and observed a statistical decrease. However, adding puerarin increased PRL levels in postpartum hypogalactia mice, suggesting that puerarin may act on the pituitary to regulate PRL secretion.

PRLR, a single-chain transmembrane protein, is essential for PRL-triggered subsequent signal transduction and gene transcription (33). After binding to PRL, the PRLR dimer initiates a conformation change, activating the JAK2 kinase proteins linked to these receptors (34). The activated JAK2 then phosphorylates STAT5 proteins, which translocate to the nucleus to induce the transcription of target genes (34). The JAK-STAT signaling pathway plays a crucial role in regulating mammary gland development and lactation. Zhang *et al*.’s study showed that the raw Hordei Fructus Germinatus effectively enhances the lactation of rats via the JAK/STAT signaling pathway (35). Geng *et al*. also demonstrated that PRL can up-regulate β-casein expression by activating the JAK2-STAT5 pathway (36). This study showed that the expression level of PRLR in the agalactosis model group was significantly decreased. On the contrary, the additional puerarin treatment substantially increased the protein expression of PRLR. The expression trends of JAK2 and STAT5A were the same as those of PRLR, suggesting that puerarin may promote lactation through the PRLR/JAK2/STAT5 signaling pathway.

However, this study has several limitations. For instance, the effects and mechanisms of different doses of puerarin on lactation need to be explored further. Additionally, the long-term safety of puerarin for both parental mice and their offspring should be further validated.

Forty mice were randomly divided into five groups: control group (CON), agalactosis model group (MOD), domperidone group (DOM), low-dose puerarin group (LPUE), and high-dose puerarin group (HPUE). From day 3 post-delivery, all groups except the control group received bromocriptine (1.6 mg/kg) intragastrically at 9:00 AM for ten days to establish the agalactosis model. Concurrently, the treatment groups received intragastric administration of domperidone (3.5 mg/kg), low-dose puerarin (18 mg/kg), or high-dose puerarin (72 mg/kg) at 2:00 PM for 10 days. The control and agalactosis model groups received an equal volume of normal saline.

A: Comparison of the average milk yield among five groups during 10 days. B: Average weight gains of pups in each group. C: Average weight loss of postpartum mice in each group. D: Changes in mammary gland weights of postpartum mice. E: Mammary gland relative weight and breasts appearance on the abdomen of mice. F: Subcutaneous distribution of breast tissues. G: Breast tissue histopathological examinations (100 and 400 magnification, H&E stained section).

Note: Group differences were analyzed using ANOVA, followed by the LSD test. The data are presented as the mean ± SD; n= 8. *Statistically different from the control group, *P*<0.05. **Statistically different from the control group, *P*<0.01. #Statistically different from the model group, *P*<0.05. ##Statistically different from the model group, *P*<0.01. CON: the control group; MOD: the agalactosis model group; DOM: the domperidone group; LPUE: the low-dose puerarin group; HPUE: the high-dose puerarin group.

A: Western blotting analysis of β-casein. B: Relative β-casein level determined by western blotting analysis. C: Western blotting analysis of FAS and GLUT1. D-E: Relative FAS and GLUT1 levels were determined using western blotting assay.

Note: Figure 4B and Figure 4D Group differences were analyzed using the Kruskal-Wallis test, followed by Bonferroni correction. Group differences of Figure 4E were analyzed by ANOVA, followed by the LSD test. The data are presented as the mean ± SD or median ± IQR; n= 3. *Statistically different from the control group, *P*<0.05. **Statistically different from the control group, *P*<0.01. #Statistically different from the model group, *P*<0.05. ##Statistically different from the model group, *P*<0.01. β-casein: beta casein; GLUT1: glucose transporter 1; FAS: fatty acid synthase. CON: the control group; MOD: the agalactosis model group; DOM: the domperidone group; LPUE: the low-dose puerarin group; HPUE: the high-dose puerarin group.

A-B: PRL levels of postpartum mice were detectable in serum and mammary gland tissues. C-D: Serum E2 and P4 levels among the five groups of postpartum mice

Note: Figure 5A and 5B Group differences were analyzed using ANOVA, followed by an LSD test. Figure 5C, 5D group differences were analyzed by the Kruskal-Wallis test, followed by Bonferroni correction. The data are presented as the mean ± SD or median ± IQR; n= 8. *Statistically different from the control group,* P*<0.05. **Statistically different from the control group, *P*<0.01. ##Statistically different from the model group, *P*<0.01. PRL: prolactin; E2: estradiol; P4: progesterone. The value of Figure 5B represents the content of PRL in the supernatant of breast tissue after treatment following the instructions provided by the ELISA kit. CON: the control group; MOD: the agalactosis model group; DOM: the domperidone group; LPUE: the low-dose puerarin group; HPUE: the high-dose puerarin group.

A: Bands of western blotting analysis for PRLR. B: Relative PRLR level was determined by western blotting assay. C: Bands of western blotting analysis for JAK2 and STAT5a. D-E: Relative JAK2 and STAT5a levels were determined by Western blotting assay.

Notes: Group differences were analyzed by ANOVA, followed by the LSD test. The data are presented as the mean ± SD; n= 3. *Statistically different from the control group, *P*<0.05. **Statistically different from the control group,* P*<0.01. ##Statistically different from the model group, *P*<0.01. PRLR: Prolactin receptor; JAK2: Janus kinase 2; STAT5a: Signal transduction and activator 5a. CON: the control group; MOD: the agalactosis model group; DOM: the domperidone group; LPUE: the low-dose puerarin group; HPUE: the high-dose puerarin group.

## Conclusion

The present results revealed that puerarin efficiently improved lactation yield and milk quality in milk-deficient mice. As a phytoestrogen, puerarin probably achieves this via its ability to stimulate PRL secretion and activate the PRLR/JAK2/STAT5 signaling pathway, thus promoting lactation. Consequently, puerarin could serve as a novel natural galactagogue to address the challenge of insufficient milk supply in lactating mothers. 

## Data Availability

Not applicable.

## Declaration

We acknowledge the use of Wordadvice AI to improve the language and readability of the manuscript.
